# Supplementary material for: Bacteroides uniformis Ameliorates Carbohydrate and Lipid Metabolism Disorders in Diabetic Mice by Regulating Bile Acid Metabolism via the Gut–Liver Axis
Source: Pharmaceuticals (Basel). 2024 Aug 1;17(8):1015. doi: 10.3390/ph17081015 (PMC11357665; doi:10.3390/ph17081015)
Supplement: Supplementary file 1 [file pharmaceuticals-17-01015-s001.zip › pharmaceuticals-3043555-supplementary.pdf]

Supplementary material and data

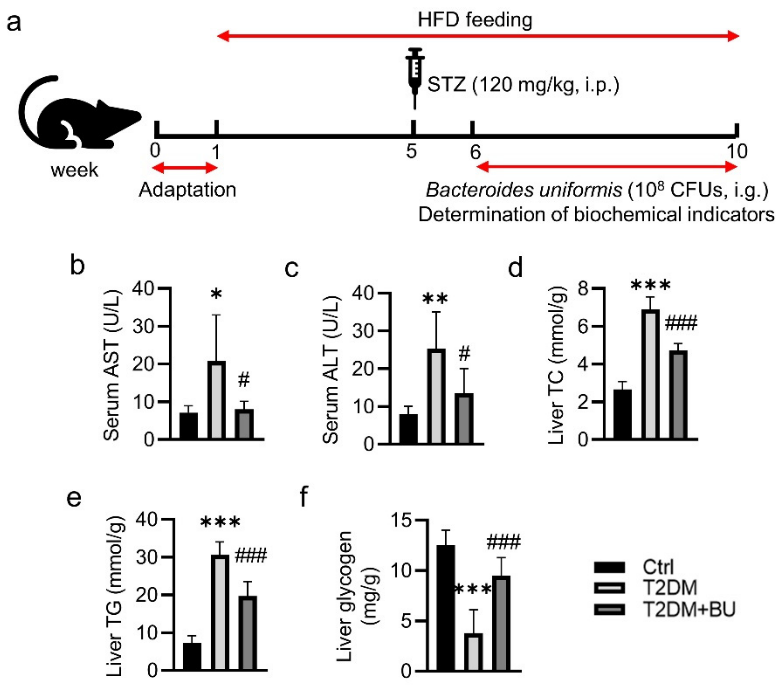

Supplementary Figure S1. (a) Experimental protocol. **(b-c)** The levels of serum AST, ALT. **(d-f)** The content of liver TC, TG and glycogen. Data were represented as mean ± SD (n=6). *P < 0.05, **P < 0.01, ***P < 0.001 vs Ctrl; #P < 0.05, ###P < 0.001 vs T2DM. Ctrl, control; T2DM, type 2 diabetes mellitus; BU, *Bacteroides uniformis*; AST, Aspartate aminotransferase; ALT, Alanine aminotransferase; TC, total cholesterol; TG, triglycerides.

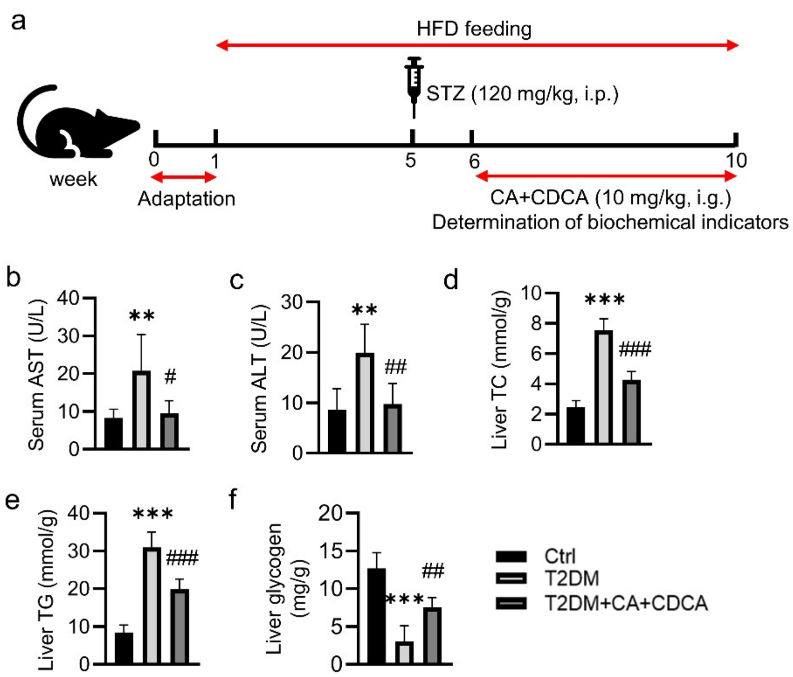

Supplementary Figure S2. (a) Experimental protocol. **(b-c)** The levels of serum AST, ALT. **(d-f)** The content of liver TC, TG and glycogen. Data were represented as mean ± SD (n=6). **P < 0.01, ***P < 0.001 vs Ctrl; #P < 0.05, ##P < 0.01, ###P < 0.001 vs T2DM. Ctrl, control; T2DM, type 2 diabetes mellitus; STZ, streptozocin; HFD, high-fat diet; AST, Aspartate aminotransferase; ALT, Alanine aminotransferase; TC, total cholesterol; TG, triglycerides; CA, cholic acid; CDCA, chenodeoxycholic acid.

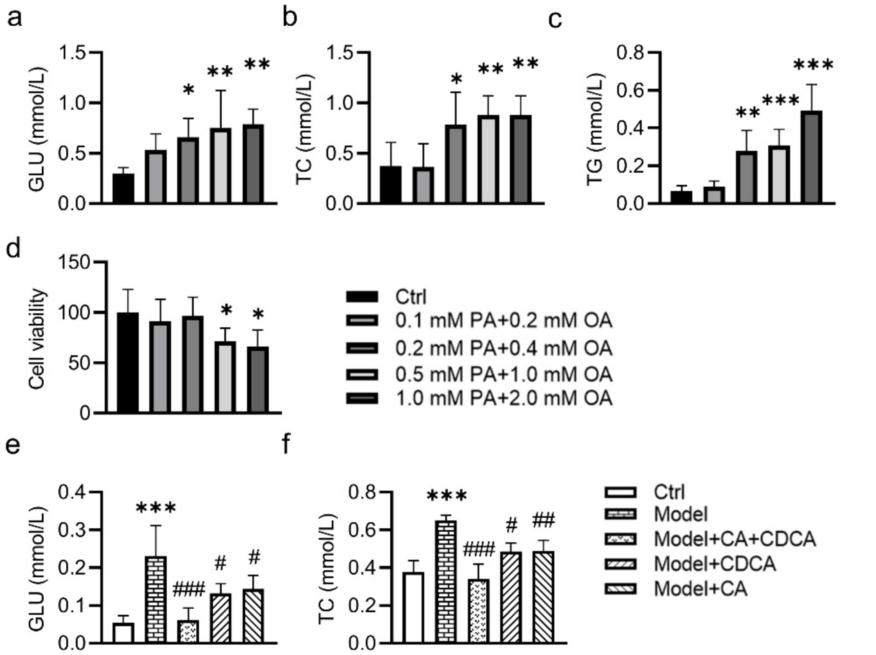

Supplementary Figure S3. Effect of different concentrations of CA and CDCA on PA/OA on insulin resistance and lipid deposition. (**a-d**) HepG2 cells were treated with different concentrations of PA and OA for 24 h. The levels of GLU, TC and TG were determined. Cell viability was measured by CCK-8 assay. (**e-f**) HepG2 cells were pre-incubated with 50 μM CA, CDCA or, CA and CDCA for 30 min, and then treated without or with 0.5 mM of PA and 1 mM of OA for 24 h. The levels of GLU and TC were measured. Data were represented as mean ± SD (n=3-6). *$P < 0.05$, **$P < 0.01$, ***$P < 0.001$ vs Ctrl; $^{\#}P < 0.05$, $^{\#\#}P < 0.01$, $^{\#\#\#}P < 0.01$ vs Model. PA, palmitic acid; OA, oleic acid; GLU, glucose; TC, total cholesterol; TG, triglycerides; CA, cholic acid; CDCA, chenodeoxycholic acid.

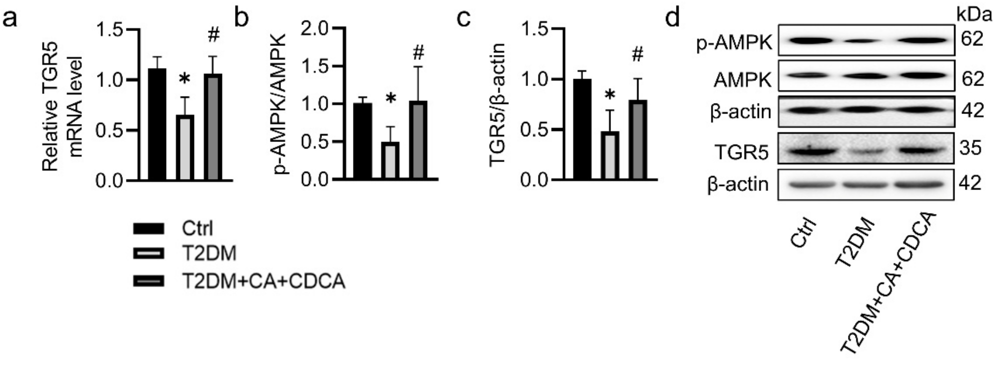

Supplementary Figure S4. Effect of CA and CDCA on TGR5/cAMP/AMPK signaling pathway in T2DM mice. (**a**) The relative mRNA levels of TGR5. (**b-d**) Representative blots and quantification of p-AMPK/AMPK and TGR5. Data were represented as mean ± SD (n =3-6). **P* < 0.05, ***P* < 0.01 vs Ctrl; $^{\#}P < 0.05$ vs T2DM. Ctrl, control; T2DM, type 2 diabetes mellitus; TGR5, Takeda G protein-coupled receptor 5; AMPK, adenosine monophosphate activated protein kinase; p-AMPK, phosphorylated adenosine monophosphate activated protein kinase; CA, cholic acid; CDCA, chenodeoxycholic acid.

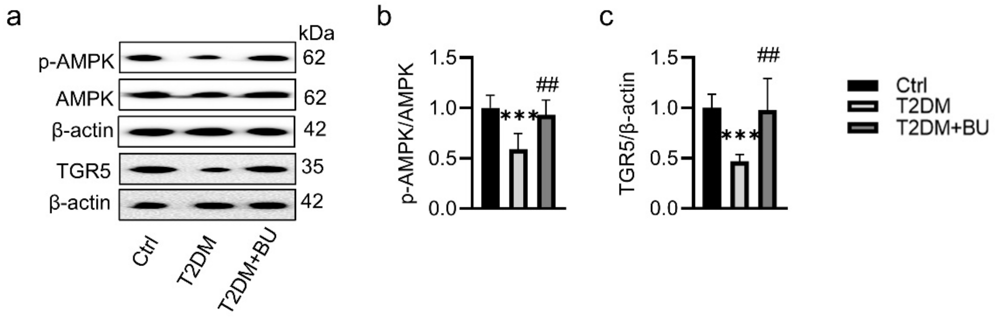

Supplementary Figure S5. Effect of CA and CDCA on TGR5/cAMP/AMPK signaling pathway in T2DM mice. (**a**) Representative blots of p-AMPK/AMPK and TGR5. (**b-c**) Quantification of p-AMPK/AMPK and TGR5. Data were represented as mean ± SD (n = 6). ****P* < 0.01 vs Ctrl; $^{\#}P < 0.05$ vs T2DM. Ctrl, control; T2DM, type

2 diabetes mellitus; BU, *B. uniformis*; TGR5, Takeda G protein-coupled receptor 5; AMPK, adenosine monophosphate activated protein kinase; p-AMPK, phosphorylated adenosine monophosphate activated protein kinase.

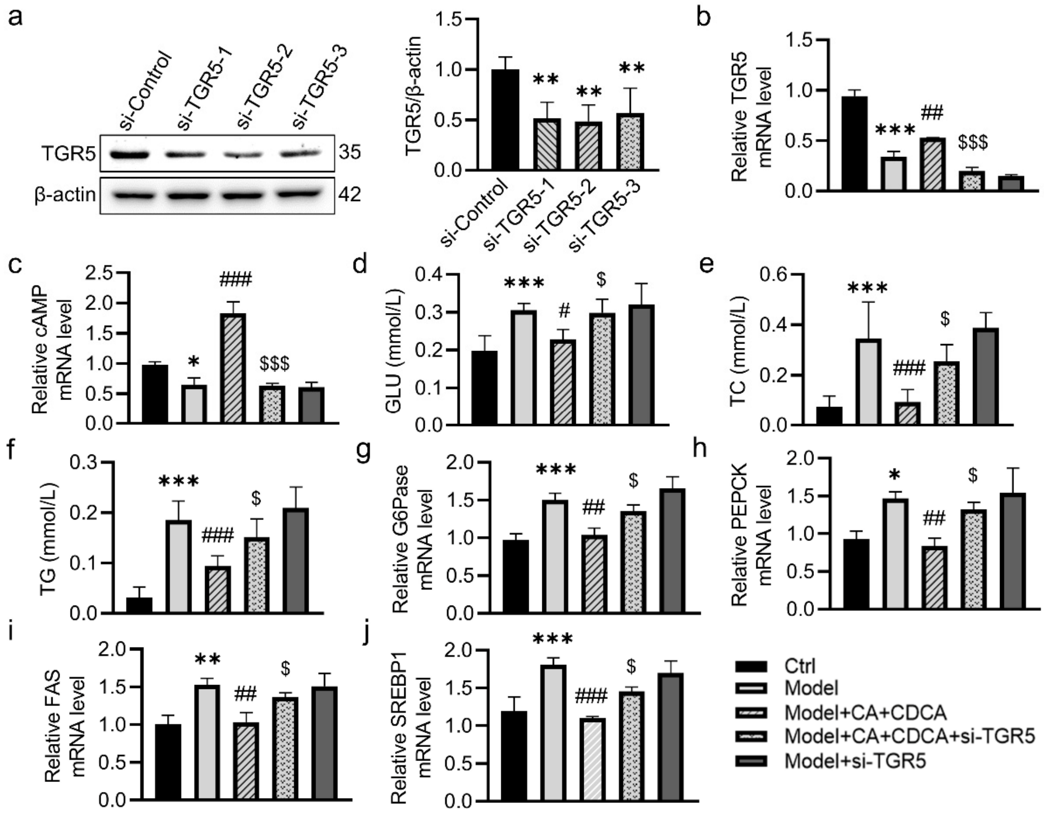

Supplementary Figure S6. Deficiency of TGR5 prevented the effects of CA and CDCA on carbohydrate and lipid metabolism. (**a**) Representative blots and quantification of TGR5. (**b**) The relative mRNA levels of TGR5. (**c-e**) The content of GLU, TC and TG. (**f-i**) The mRNA levels of G6Pase, PEPCK, FAS, and SREBP1. Data were represented as mean ± SD (n =3-6). **P* < 0.05, ***P* < 0.01, ****P* < 0.001 vs Ctrl; $^{\#}P < 0.05$, $^{\#\#}P < 0.01$, $^{\#\#\#}P < 0.01$ vs Model. $^{\$}P < 0.05$, $^{\$\$\$}P < 0.01$ vs Model+CA+CDCA. Ctrl, control; CA, cholic acid; CDCA, chenodeoxycholic acid; TGR5, Takeda G protein-coupled receptor 5; GLU, glucose; TC, total cholesterol; TG,

triglycerides; G6Pase, glucose-6-phosphatase; PEPCK, phosphoenolpyruvate carboxykinase; FAS, fatty acid synthase; SREBP1, sterol-regulatory element binding protein 1.

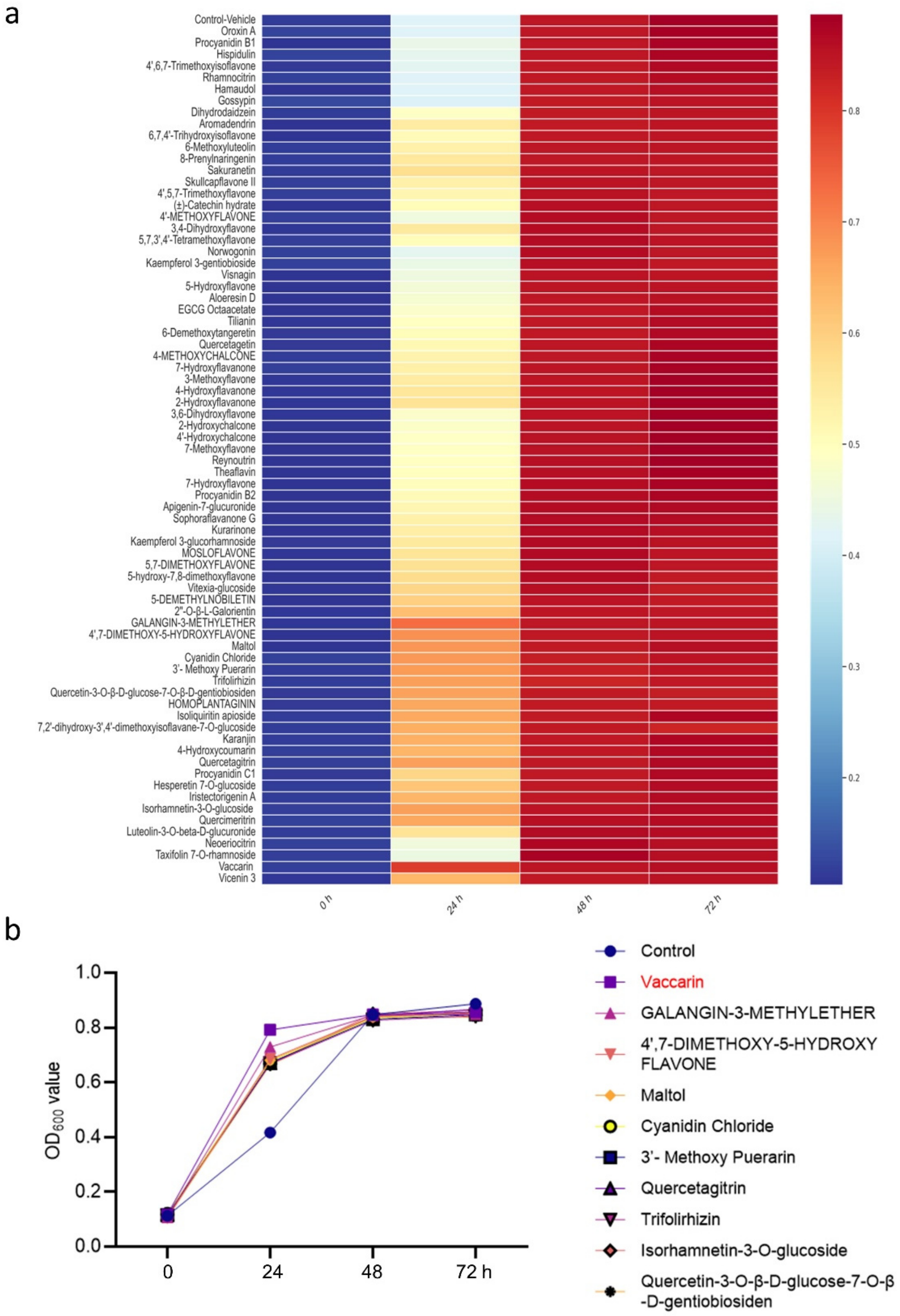

Supplementary Figure S7. Effect of different natural flavonoids on the growth of *B. uniformis*. (**a**) Heatmap showing the effects of 79 compounds (10 μM) on the growth of *B. uniformis in vitro*, as assessed by OD_{600} value. (**b**) The growth curve of *B. uniformis* induced by top-ten saponins at indicated time points.

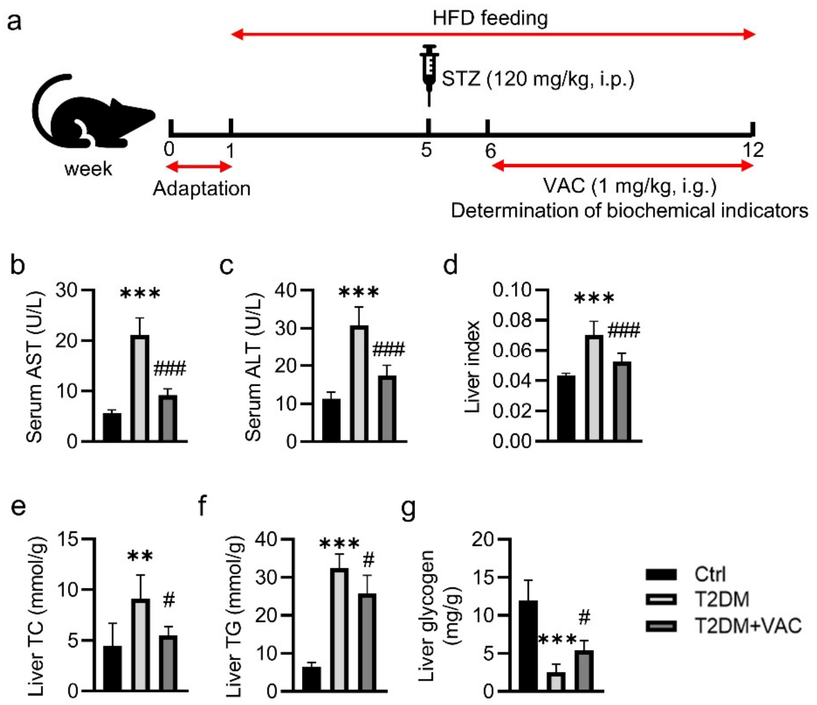

Supplementary Figure S8. (a) Experimental protocol. **(b-c)** The levels of serum AST, ALT. **(d)** The liver index. **(e-g)** The content of liver TC, TG and glycogen. Data were represented as mean ± SD (n=6). **P < 0.01, ***P < 0.001 vs Ctrl; #P < 0.05, ###P < 0.001 vs T2DM. Ctrl, control; T2DM, type 2 diabetes mellitus; VAC, vaccarin; AST, Aspartate aminotransferase; ALT, Alanine aminotransferase; TC, total cholesterol; TG, triglycerides.

Table S1. Primer sequences used for measurement of the bacteria in the gut.

Species	Gene	Sequence
Mice	β-actin	F: 5'-AGCTGAGAGGGAAATCGTGC-3'<br>R: 5'-TCCAGGGAGGAAGAGGATGC-3'
Mice	*B. uniformis*	F: 5'-TAGCGGTGAAATGCTTAG-3'<br>R: 5'-CATCGTTTACTGTGTGGA-3'

Table S2. The primer sequences used in HepG2 cells.

Gene	Forward primer (5′->3′)	Reverse primer (5′->3′)
β-actin	ATCATGTTTGAGACCTTCAACA	CATCTCTTGCTCGAAGTCCA
G6Pase	ACTGGCTCAACCTCGTCTTTA	CGGAAGTGTTGCTGTAGTAGTCA
PEPCK	GAAAAAACCTGGGGCACAT	TTGCTTCAAGGCAAGGATCTCT
FAS	AGCTGCCAGAGTCGGAGAAC	GTAGCCCACGAGTGTCTCG
SREBP1	ACAGTGACTTCCCTGGCCTAT	GCATGGACGGGTACATCTTCAA
TGR5	CTGCTGGCTGCTTCTTCCTGAG	ACGAGGAGGCAGGACCAGTAAC

Table S3. The primer sequences used in mice.

Gene	Forward primer (5′->3′)	Reverse primer (5′->3′)
β-actin	AGCTGAGAGGGAAATCGTGC	TCCAGGGAGGAAGAGGATGC
G6Pase	CCGGATCTACCTTGCTGCTC	GCATTGTAGATGCCCCGGAT
PEPCK	TGGAAGGTCGAATGTGTGGG	CAGTAAACACCCCCATCGCT
FAS	TGCTTGCTGGCTCACAGTTAAGAG	TTTCACGAACCCGCCTCCTCAG
SREBP1	GGATGCGGCTGTTGTCTACCATAAG	CCAGGTTAGAAGCAGCAAGATGTCC
TGR5	GCCTGGAACTCTGTTATCGCTCATC	GAAGCACTCGTAGACACCTTTGGG
